# Explainable AI identifies key biomarkers for acute kidney injury prediction in the ICU

**DOI:** 10.1186/s40635-025-00816-x

**Published:** 2025-10-22

**Authors:** Hazem Koozi, Jonas Engström, Hans Friberg, Attila Frigyesi

**Affiliations:** 1 Department of Clinical Sciences, Anaesthesiology and Intensive Care, Lund University, SE-22185 Lund, Sweden; 2 Department of Anaesthesia and Intensive Care, Skåne University Hospital, SE-29133 Kristianstad, Sweden; 3 Department of Intensive and Perioperative Care, Skåne University Hospital, SE-20502 Malmö, Sweden; 4 Department of Intensive and Perioperative Care, Skåne University Hospital, SE-22185 Lund, Sweden

**Keywords:** Intensive care, Biomarkers, Acute kidney injury, Renal replacement therapy, Artificial intelligence

## Abstract

**Background:**

Early identification of acute kidney injury (AKI) in the intensive care unit (ICU) remains challenging. We aimed to identify key predictors of new-onset AKI within 48 h after ICU admission and renal replacement therapy (RRT) need within 7 days, using explainable artificial intelligence (XAI) with eXtreme Gradient Boosting (XGBoost). We also assessed whether XGBoost improved predictive performance.

**Methods:**

A retrospective cohort study across four ICUs was conducted as part of the SWECRIT biobank project. Blood samples were prospectively obtained at ICU admission and retrospectively analysed. AKI was defined by the Kidney Disease: Improving Global Outcomes (KDIGO) criteria. XAI models were compared with logistic regression models, incorporating emerging biomarkers and routine clinical data at ICU admission. SHapley Additive exPlanations (SHAP) were used to identify key predictors. Discrimination was assessed using the mean area under the receiver operating characteristic curve (AUC).

**Results:**

The study included 4732 admissions, with 2603 analysed for new-onset AKI and 4716 for RRT. Top predictors of new-onset AKI were urine output, endostatin, baseline creatinine, lactate, and albumin. Top predictors of RRT were creatinine, urine output, endostatin, neutrophil gelatinase-associated lipocalin (NGAL), and the Simplified Acute Physiology Score (SAPS) 3. Several clinically relevant non-linear relationships were revealed. XGBoost outperformed logistic regression for both new-onset AKI (mean AUC 0.76, 95% CI 0.70–0.81 vs. 0.74, 95% CI 0.68–0.81; *p* < 0.001) and RRT (mean AUC 0.92, 95% CI 0.89–0.95 vs. 0.90, 95% CI 0.87–0.94; *p* < 0.001).

**Conclusion:**

XGBoost identified key predictors of early new-onset AKI and RRT need in the ICU, highlighting both emerging (endostatin, NGAL) and established biomarkers (lactate, albumin), alongside known clinical predictors. It also improved predictive accuracy for both outcomes. Further clinical evaluation of these biomarkers and XAI models is warranted.

**Supplementary Information:**

The online version contains supplementary material available at 10.1186/s40635-025-00816-x.

## Background

Acute kidney injury (AKI) is a common and multifactorial complication of critical illness, associated with substantial morbidity and mortality. It affects approximately 50% of intensive care unit (ICU) admissions [1]. Creatinine and urine output, the most widely used biomarkers of AKI, are suboptimal. Significant glomerular filtration rate (GFR) loss occurs before creatinine increases [2]. Furthermore, creatinine levels rise slowly and are influenced by muscle mass, while oliguria may reflect a transient physiological response or be affected by diuretics [3].

Early detection of AKI is essential, enabling timely nephroprotective measures. Delayed identification of AKI and the condition’s heterogeneity are major factors contributing to the lack of effective therapies. Emerging biomarkers hold promise for improving early recognition of AKI. However, despite extensive research into many promising biomarker candidates, few have been adopted in clinical practice [3].

Machine learning (ML) techniques are increasingly used in medicine to analyse complex data and detect patterns beyond traditional statistics. Among these, eXtreme Gradient Boosting (XGBoost) is a powerful algorithm that sequentially builds an ensemble of decision trees, thereby improving predictive accuracy, reducing overfitting, and facilitating the identification of key predictors [4]. SHapley Additive exPlanations (SHAP) is an explainable artificial intelligence (XAI) method that quantifies the contribution of each predictor to an ML model, enabling the interpretation of complex models like XGBoost [5].

Two prior studies have demonstrated the utility of XGBoost for AKI prediction in a general ICU population [6, 7]. However, integrating XGBoost with emerging biomarkers in intensive care has not yet been explored.

### Objectives

We aimed to identify the most important predictors of new-onset AKI within 48 h after ICU admission and the need for renal replacement therapy (RRT) within 7 days, using an XAI approach based on XGBoost. In addition, we evaluated whether XGBoost could improve predictive performance compared to logistic regression.

## Methods

### Study design and setting

We conducted a retrospective multicentre cohort study as part of the larger SWECRIT biobank project (ClinicalTrials.gov ID: NCT04974775, retrospectively registered on June 18, 2021) [8]. Consecutive ICU admissions between 2015 and 2018 were included from four general ICUs in southern Sweden: Skåne University Hospital in Lund and Malmö, Helsingborg Hospital, and Kristianstad Hospital. Blood samples were prospectively obtained at ICU admission and preserved in the SWECRIT biobank for later retrospective analyses. The Strengthening the Reporting of Observational Studies in Epidemiology (STROBE) guidelines were followed [9].

### Participants

All adult ICU admissions were screened for eligibility. Exclusion criteria included ICU discharge alive within 24 h, incorrect handling of the biobank sample, withdrawal of consent, and ICU transfer without resampling.

### Variables

The selection of biomarkers was based on prior evidence and data availability. Cystatin C, neutrophil gelatinase-associated lipocalin (NGAL), endostatin, intercellular adhesion molecule 1 (ICAM-1), vascular cell adhesion molecule 1 (VCAM-1), and albumin were included due to their previously reported associations with AKI, whereas C-reactive protein (CRP) and calprotectin were primarily analysed for another SWECRIT biobank study and were, therefore, incorporated as they were available [10–15]. All other variables used in the XGBoost and logistic regression analyses were included based on their availability in the data set.

Creatinine, cystatin C, NGAL, endostatin, ICAM-1, VCAM-1, albumin, CRP, and calprotectin in plasma were retrospectively batch-analysed from prospectively collected blood samples obtained on ICU admission. Creatinine was also analysed on the first 2 days of the ICU stay or until ICU discharge, whichever occurred first. For baseline creatinine, the value closest to ICU admission within 7 to 365 days before was recorded. If baseline creatinine was missing, it was estimated using the 2021 Chronic Kidney Disease–Epidemiology Collaboration (CKD–EPI) creatinine equation, assuming a baseline estimated glomerular filtration rate (eGFR) of 75 mL/min/1.73 $\textrm{m}^2$ for AKI classification [16]. Chronic kidney disease (CKD) was defined as eGFR < 60 mL/min/1.73 $\textrm{m}^2$ [17]. Urine output was recorded at ICU admission and over the first two ICU days or until discharge. If urine output could not be monitored for 24 h, it was estimated by extrapolating from hourly urine output. Urine output at ICU admission was not used for AKI classification. Instead, urine output was incorporated into AKI classification on ICU days 1 and 2. AKI was defined as fulfilment of the Kidney Disease: Improving Global Outcomes (KDIGO) criteria within 48 h after ICU admission [17]. New-onset AKI was defined as the absence of AKI at ICU admission, followed by its subsequent development within 48 h after ICU admission. Admissions with AKI or missing AKI status on ICU admission were excluded from analyses of new-onset AKI. RRT was regarded as AKI unless the admission had a diagnosis of dialysable intoxication, in which case, they needed to fulfil another KDIGO criterion. For RRT, we evaluated its initiation in the ICU within 7 days after ICU admission.

For variables included in the Simplified Acute Physiology Score (SAPS) 3 and the Sequential Organ Failure Assessment (SOFA) (excluding admission creatinine), the worst recorded values within an hour of ICU admission were used [18, 19]. The value closest in time, within 24 h, of ICU admission was recorded for lactate and white blood cell (WBC) count.

Immunosuppressive treatment, metastatic and haematological cancer, cirrhosis, and chronic heart failure were defined according to SAPS 3 [18].

Sepsis was defined according to both the Sepsis-3 criteria and the Linder–Mellhammar Criteria of Infection [19, 20].

ICU day 1 was defined as beginning at 6 a.m. on the morning following ICU admission, with ICU day 2 defined accordingly.

### Data sources

Blood samples were collected using ethylenediamine tetraacetic acid (EDTA) vacutainers and centrifuged to obtain EDTA plasma. The plasma samples were aliquoted and stored in the SWECRIT biobank at −80 $^{\circ }$C. Samples had to be collected within 6 h of ICU admission. If the sampling time was missing, samples were included if the freezing time fell within the 6 h time frame.

Admission creatinine and cystatin C were analysed on a Mindray BS380 chemistry analyser (Mindray Medical International, Shenzhen, China) using isotope dilution mass spectrometry traceable enzymatic creatinine reagents from Abbott Laboratories (Abbott Park, IL, USA) and particle-enhanced turbidimetric cystatin C reagents from Gentian (Moss, Norway). Creatinine at baseline and on ICU days 1 and 2 was also analysed using the enzymatic method. NGAL and endostatin analyses were performed using commercial sandwich enzyme-linked immunosorbent assay (ELISA) kits (DY1757/DY1098, R&D Systems, Minneapolis, MN, USA). ICAM-1 and VCAM-1 were also analysed using commercial sandwich ELISA kits (DY720/DY809, R&D Systems). Albumin was measured on a Mindray BS380 chemistry analyser with reagents from Abbott Laboratories. For the analysis of CRP and calprotectin, a particle-enhanced turbidimetric immunoassay methodology was used with CRP reagents from Abbott Laboratories and calprotectin reagents from Gentian AS on a Mindray BS430/BS380 chemistry analyser.

Body weight, body mass index (BMI), diabetes mellitus, hypertension, and chronic dialysis were manually collected from electronic medical records. All other clinical data were automatically extracted from electronic medical records.

### Study size

The sample size was determined by the number of ICU admissions during the study period, ICU length of stay, the validity of the collected blood samples, and patient consent. For the analysis of new-onset AKI, the sample size was further reduced by excluding admissions who had AKI at ICU admission. For the RRT analysis, missing data on RRT start time contributed to a reduction in the number of included admissions.

### Bias

Treating clinicians and data collectors were unfamiliar with NGAL, endostatin, ICAM-1, VCAM-1, and calprotectin levels. However, they had access to creatinine levels, and occasionally cystatin C and albumin, as part of routine intensive care. Knowledge of these levels may have influenced clinical decisions. Trained data collectors performed the manual data recording. Guidelines for data collection were standardised and precisely outlined. The handling of missing data (e.g., the use of imputation) was discussed and decided on collectively by the study authors.

### Statistics

Statistical analyses were performed in R [21]. Significance was determined as a *p*-value of less than 0.05.

The Wilcoxon rank-sum test was used to compare medians, Pearson’s chi-squared test for proportions, and the unpaired *t* test for means.

In cases where body weight was missing, body weight was imputed using linear regression based on age and sex, and the imputed value was subsequently used to estimate urine output normalisation for AKI classification.

For logistic regression and XAI analyses, variables with a missing rate of more than 20% were excluded, except for baseline creatinine. Missing values in the remaining variables were imputed using a random forest–based method in two iterations using 100 trees.

Continuous variables with non-normal distributions were log-transformed before logistic regression and XAI analyses.

For XAI analyses, the XGBoost package (version 1.7.9.1) was used [4]. Limited hyperparameter tuning by grid search was performed (boosting rounds of 100–300, maximum tree depth of 3–5, learning rate of 0.03–0.05, gamma of 0–1, and minimum child weight of 3–5). The final XGBoost model used 300 boosting rounds, a maximum tree depth of 3, a learning rate of 0.03, a gamma of 1, a subsample of 0.8, a column sample by tree of 0.8, and a minimum child weight of 5 for both outcomes. Logistic regression was utilised alongside XGBoost to create prediction models of new-onset AKI and RRT. Model training and performance evaluation were conducted using repeated tenfold cross-validation with 20 repetitions.

SHAP values were calculated to assess the relative importance of each variable and the direction of its association within the XGBoost models. SHAP values estimate each variable’s contribution to the prediction for a given patient while accounting for the influence of all other model variables. A positive value indicates that the variable increases the predicted risk, whereas a negative value indicates that it lowers the predicted risk [5]. SHAP summary plots with mean SHAP values were utilised to illustrate results across the entire cohort: predictors were ranked by their overall influence, and each dot represented one patient. The dot’s colour showed whether the variable value was low or high, and its position on the *x*-axis indicated whether that value increased or decreased the risk. In addition, SHAP dependence plots were used to visualise the relationship between individual predictor variables and their contribution to the model’s output, allowing assessment of non-linear effects and interactions.

Model discrimination was assessed using the mean area under the receiver operating characteristic curve (AUC) with 95% confidence intervals (CI) derived from repeated cross-validation. Mean AUCs were compared using DeLong’s method [22].

## Results

### Participants

**Fig. 1:**
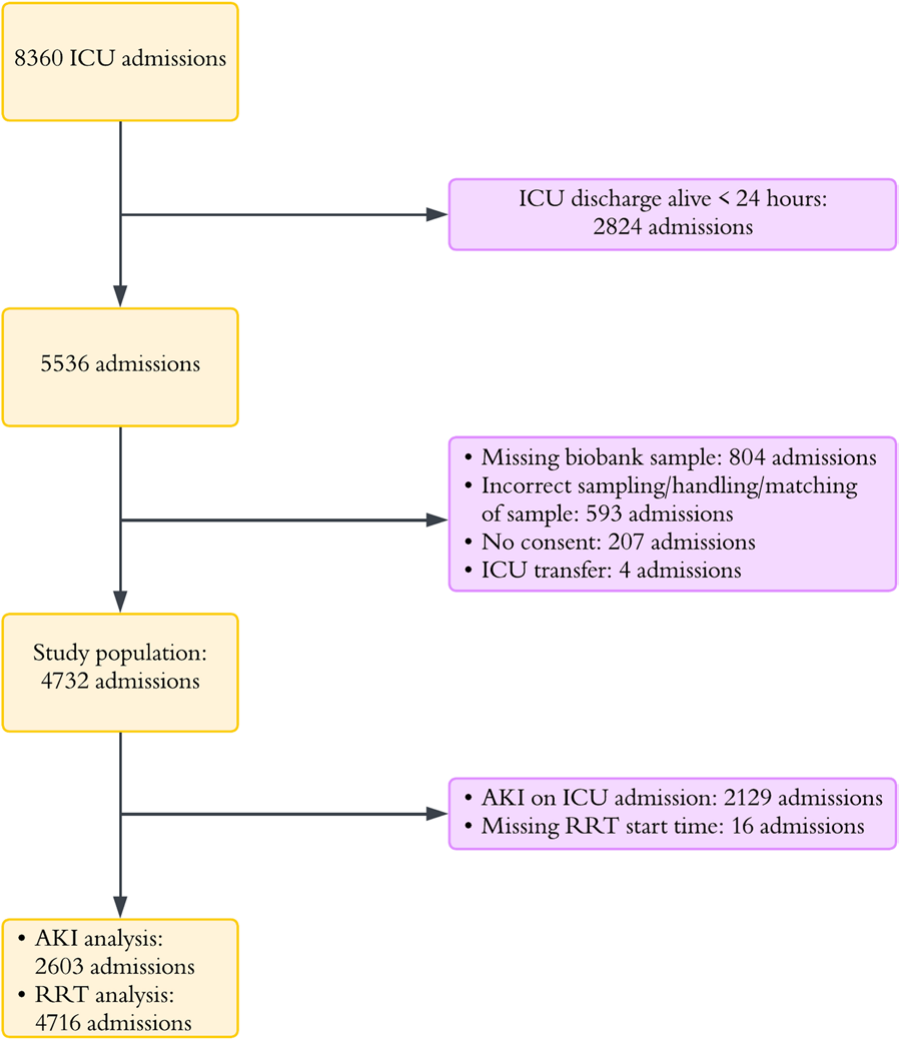
Flow chart of included and excluded ICU admissions. *ICU* Intensive Care Unit, *AKI* Acute Kidney Injury, *RRT* Renal Replacement Therapy

Of the 8360 ICU admissions identified, 4732 were included in the study. New-onset AKI analysis was performed on 2603 admissions, and 4716 were included in the RRT analysis, see Fig. 1.

### Descriptive data

The median age was 68 years (interquartile range [IQR] 56–75), with males comprising 60%. CKD was present in 17% of patients. The rate of Sepsis-3 was 42%, the rate of cardiac arrest was 11%, and the rate of trauma was 5.4%. The three most common primary ICU diagnoses were cardiac arrest (10%), septic shock (9.9%), and respiratory failure (5.3%). The median SAPS 3 score was 64 (IQR 52–75), and the mean SOFA score was 6.9 (standard deviation [SD] 4.0). Median baseline creatinine was 79 $\upmu$mol/L (IQR 62–110), while median creatinine on ICU admission was 100 $\upmu$mol/L (IQR 69–160).

Admissions with new-onset AKI were older, had higher BMI, higher rates of sepsis and cardiac arrest, a lower rate of trauma, higher rates of comorbidities (except cancer), higher SAPS 3 and SOFA scores, lower blood pressure, and a higher rate of vasopressor therapy compared to admissions without new-onset AKI. They had higher levels of baseline and admission creatinine, cystatin C, NGAL, endostatin, ICAM-1, VCAM-1, bilirubin, and lactate. Admissions with new-onset AKI also had lower levels of albumin and platelets.

Admissions with RRT were also older, had higher BMI, a higher rate of sepsis, lower rates of cardiac arrest and trauma, higher rates of comorbidities (except immunosuppressive therapy and cancer), higher SAPS 3 and SOFA scores, lower blood pressure, a higher rate of vasopressor therapy, and higher Glasgow Coma Scale compared to admissions without RRT. They had higher levels of baseline and admission creatinine, cystatin C, NGAL, endostatin, ICAM-1, VCAM-1, CRP, calprotectin, bilirubin, and lactate. Admissions with RRT also had lower levels of albumin and platelets.

See Tables 1 and 2 for descriptive data based on new-onset AKI and RRT status.

**Table 1:** Characteristics of the study population divided into new-onset AKI status

	No AKI	AKI	*p*-value	Missing (%)
*n* (%)	1581 (61)	1022 (39)		
General characteristics				
Age (years)	64 (48–72)	70 (60–76)	< 0.001	0
Male sex (%)	57	61	0.61	0
Body mass index (kg/m^2^)	26 (22–30)	27 (24–32)	< 0.001	65
Sepsis-3 (%)	34	38	0.021	0
Cardiac arrest (%)	8.9	13	0.0022	2.7
Trauma (%)	8.9	5.3	< 0.001	1.4
Time before ICU (days)	0 (0–2.0)	0 (0–3.0)	0.031	0
Comorbidities				
Immunosuppressive therapy (%)	6.4	11	< 0.001	0
Metastatic cancer (%)	12	12	0.79	0
Haematological cancer (%)	2.0	2.3	0.80	0
Cirrhosis (%)	0.95	2.3	0.0068	0
Chronic heart failure (%)	5.6	11	< 0.001	0
Hypertension (%)	13	21	< 0.001	49
Diabetes mellitus (%)	6.2	10	< 0.001	62
Chronic kidney disease (%)	8.9	25	< 0.001	35
Chronic dialysis (%)	0	2.6	< 0.001	62
Illness severity				
SAPS 3 score	55 (45–66)	63 (53–75)	< 0.001	0
SOFA score^*^	5.1 (3.2)	7.0 (3.8)	< 0.001	4.0
PaO_2_/FiO_2_ (kPa)	31 (19–48)	26 (16–40)	< 0.001	15
Mean arterial pressure (mmHg)	70 (61–81)	65 (60–75)	< 0.001	1.7
Vasopressor therapy (%)	42	56	< 0.001	1.4
Glasgow Coma Scale^*^	12 (4.3)	11 (4.4)	0.058	2.3
Laboratory values				
Baseline creatinine ($\upmu$mol/L)	71 (58–90)	86 (66–128)	< 0.001	35
Creatinine ($\upmu$mol/L)	72 (55–89)	84 (64–110)	< 0.001	0
Cystatin C (mg/L)	0.79 (0.61–1.1)	1.0 (0.71–1.5)	< 0.001	0
NGAL (ng/mL)	130 (84–210)	190 (120–360)	< 0.001	0
Endostatin (ng/mL)	48 (38–63)	63 (48–87)	< 0.001	0
ICAM-1 (ng/mL)	180 (140–260)	200 (150–290)	< 0.001	0
VCAM-1 (ng/mL)	290 (210–400)	350 (250–500)	< 0.001	0
C-reactive protein (mg/L)	23 (3.2–100)	27 (4.5–99)	0.11	0
Calprotectin (mg/L)	1.3 (0.52–2.6)	1.3 (0.55–2.8)	0.30	0
Albumin (g/L)	25 (21–31)	23 (18–28)	< 0.001	0
Bilirubin ($\upmu$mol/L)	9 (6.0–14)	10 (6.0–17)	< 0.001	6.4
Platelet count (x10$^9$/L)	230 (170–300)	210 (150–290)	0.0024	5.8
Lactate (mmol/L)	1.5 (1.0–2.6)	2.0 (1.2–3.9)	< 0.001	0.038
Outcomes				
RRT (%)	0.063	12	< 0.001	0.23
ICU length of stay (days)	2.1 (1.5–4.0)	3.1 (1.8–6.0)	< 0.001	0
30 day mortality (%)	17	32	< 0.001	0.038

Variables are at ICU admission unless otherwise specified (except outcomes). Values are medians with interquartile ranges unless otherwise specified. *P*-values were calculated using the Wilcoxon rank-sum test and Pearson’s chi-square test as appropriate, unless otherwise specified. ^*^Presented as means with standard deviations, and *p*-value calculated using unpaired *t* test. *SAPS 3* Simplified Acute Physiology Score 3, *SOFA* Sequential Organ Failure Assessment, *PaO*_2_ Arterial Partial Pressure of Oxygen, *FiO*_2_ Fraction of Inspired Oxygen (%), *NGAL* Neutrophil Gelatinase-Associated Lipocalin, *ICAM-1* Intercellular Adhesion Molecule 1, *VCAM-1* Vascular Cell Adhesion Molecule 1, *AKI* Acute Kidney Injury, *RRT* Renal Replacement Therapy, *ICU* Intensive Care Unit

**Table 2:** Characteristics of the study population divided into RRT status

	No RRT	RRT	*p*-value	Missing (%)
n (%)	4143 (88)	573 (12)		
General characteristics				
Age (years)	68 (56–75)	70 (61–76)	< 0.001	0
Male sex (%)	60	64	0.10	0
Body mass index (kg/m^2^)	26 (23–31)	28 (24–33)	< 0.001	63
Sepsis-3 (%)	40	59	< 0.001	0
Cardiac arrest (%)	11	8.4	0.032	6.3
Trauma (%)	5.9	1.7	< 0.001	6.2
Time before ICU (days)	0 (0–2.0)	1.0 (0–6.0)	< 0.001	0
Comorbidities				
Immunosuppressive therapy (%)	8.3	9.4	0.39	0
Metastatic cancer (%)	12	9.4	0.14	0
Haematological cancer (%)	2.8	3.0	0.90	0
Cirrhosis (%)	1.7	4.7	< 0.001	0
Chronic heart failure (%)	11	8.4	0.033	0
Hypertension (%)	18	26	< 0.001	46
Diabetes mellitus (%)	9.1	19	< 0.001	58
Chronic kidney disease (%)	13	41	< 0.001	38
Chronic dialysis (%)	0.31	7.0	< 0.001	58
Illness severity				
SAPS 3 score	62 (50–74)	73 (64–82)	< 0.001	0
SOFA score^*^	6.4 (3.8)	9.9 (3.9)	< 0.001	8.6
PaO_2_/FiO_2_ (kPa)	28 (17–43)	26 (15–43)	0.096	14
Mean arterial pressure (mmHg)	65 (59–77)	60 (50–70)	< 0.001	2.9
Vasopressor therapy (%)	49	70	< 0.001	6.2
Glasgow Coma Scale^*^	11 (4.4)	12 (3.8)	0.023	2.3
Laboratory values				
Baseline creatinine ($\upmu$mol/L)	76 (61–99)	120 (79–250)	< 0.001	38
Creatinine ($\upmu$mol/L)	91 (65–140)	240 (150–470)	< 0.001	0
Cystatin C (mg/L)	1.1 (0.73–1.8)	2.7 (1.9–3.8)	< 0.001	0
NGAL (ng/mL)	190 (110–360)	640 (350–1100)	< 0.001	0
Endostatin (ng/mL)	59 (44–82)	100 (72–140)	< 0.001	0
ICAM-1 (ng/mL)	200 (150–280)	250 (180–380)	< 0.001	0
VCAM-1 (ng/mL)	340 (240–490)	540 (370–810)	< 0.001	0
C-reactive protein (mg/L)	34 (4.7–120)	87 (26–180)	< 0.001	0
Calprotectin (mg/L)	1.5 (0.66–3.2)	2.6 (1.1–5.6)	< 0.001	0
Albumin (g/L)	25 (20–30)	22 (17–27)	< 0.001	0
Bilirubin ($\upmu$mol/L)	10 (6.0–16)	14 (7.0–27)	< 0.001	6.3
Platelet count (x10$^9$/L)	210 (150–290)	170 (110–260)	< 0.001	7.8
Lactate (mmol/L)	2.0 (1.1–3.8)	2.8 (1.4–6.8)	< 0.001	0.042
Outcomes				
AKI on ICU admission (%)	35	79	< 0.001	0
New-onset AKI (%)	22	21	0.73	4.8
ICU length of stay (days)	2.3 (1.4–4.3)	4.6 (2.6–8.1)	< 0.001	0
30 day mortality (%)	29	41	< 0.001	0.021

Variables are at ICU admission unless otherwise specified (except outcomes). Values are medians with interquartile ranges unless otherwise specified. *P*-values were calculated using the Wilcoxon rank-sum test and Pearson’s chi-square test as appropriate, unless otherwise specified. ^*^Presented as means with standard deviations, and *p*-value calculated using unpaired *t* test. *SAPS 3* Simplified Acute Physiology Score 3, *SOFA* Sequential Organ Failure Assessment,*PaO*_2_ Arterial Partial Pressure of Oxygen, *FiO*_2_ Fraction of Inspired Oxygen (%), *NGAL* Neutrophil Gelatinase-Associated Lipocalin, *ICAM-1* Intercellular Adhesion Molecule 1, *VCAM-1* Vascular Cell Adhesion Molecule 1, *AKI* Acute Kidney Injury, *ICU* Intensive Care Unit

### Outcomes

The overall rate of AKI was 62% (*n* = 2922/4732), with 40% (*n* = 1900/4732) of admissions having AKI on ICU admission. New-onset AKI occurred in 22% (*n* = 1022/4732) of admissions. Among admissions with AKI, 35% (*n* = 1015/2922) had stage 1, 23% (*n* = 680/2922) had stage 2, and 42% (*n* = 1227/2922) had stage 3. The rate of missing AKI status was 4.8% (*n* = 229/4732). RRT was initiated in 14% (*n* = 661/4732) of patients during the ICU stay, and in 12% (*n* = 573/4732) within 7 days after ICU admission. The median ICU length of stay was 2.5 days (IQR 1.5–4.8). Mortality within 30 days was 30% (*n* = 1435/4732).

Admissions with new-onset AKI had a higher rate of RRT, longer ICU length of stay, and a higher 30-day mortality.

Admissions with RRT had a higher rate of AKI, longer ICU length of stay, and a higher 30-day mortality.

See Tables 1 and 2 for outcomes based on new-onset AKI and RRT status.

### Main results

**Fig. 2:**
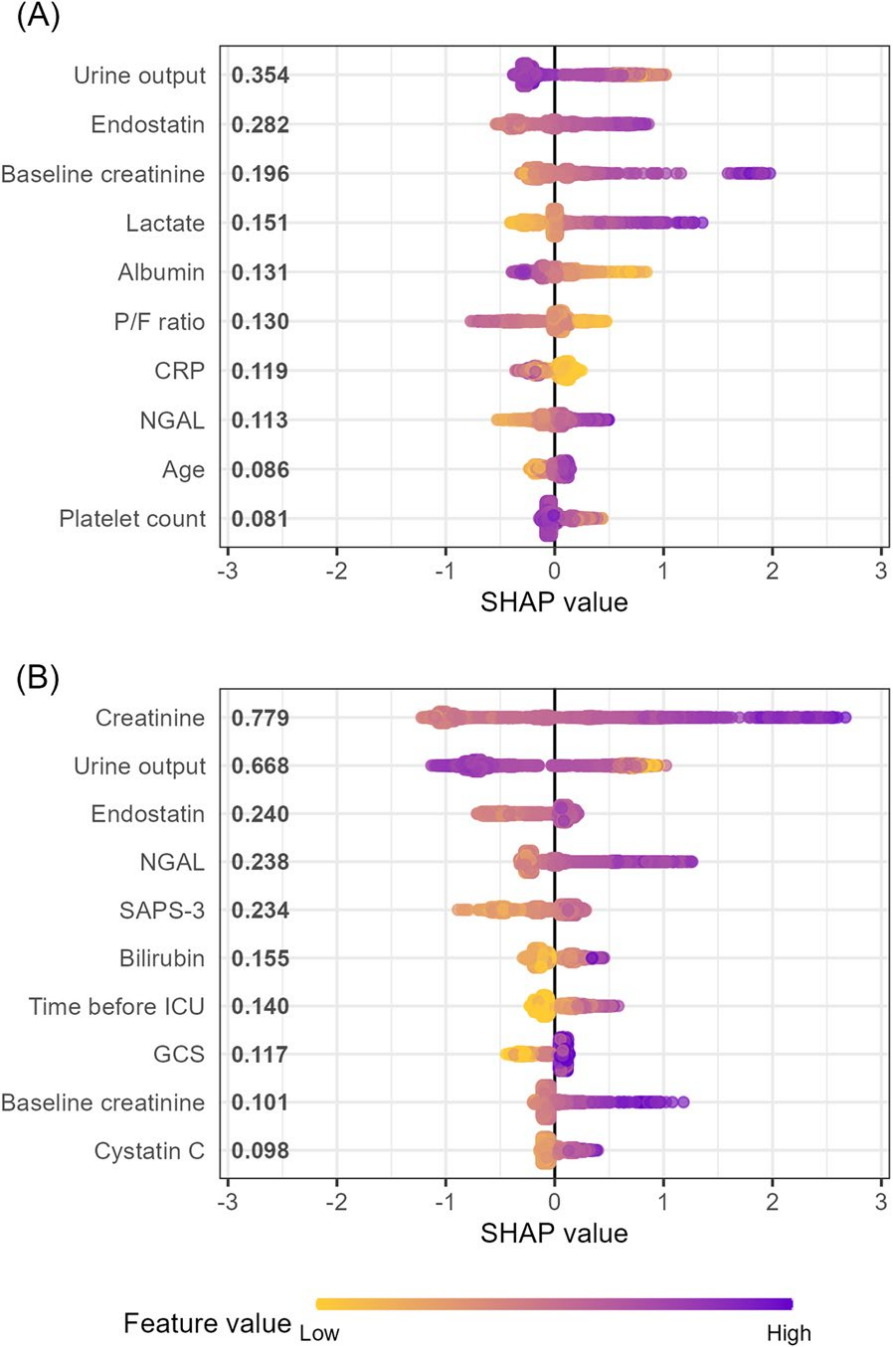
SHapley Additive exPlanations (SHAP) summary plots for eXtreme Gradient Boosting (XGBoost) models predicting new-onset AKI (**A**) and RRT (**B**). SHAP values estimate each variable’s contribution to the prediction for a given patient while accounting for the influence of all other model variables. Each plot shows the top 10 variables ranked by mean absolute SHAP values, with variable values colour-coded from low (yellow) to high (purple). Positive SHAP values indicate an increased risk, while negative values indicate a decreased risk. *AKI* Acute Kidney Injury, *RRT* Renal Replacement Therapy, *P/F ratio* PaO_2_/FiO_2_ ratio, *CRP* C-reactive protein, *NGAL* Neutrophil Gelatinase-Associated Lipocalin, *SAPS* Simplified Acute Physiology Score, *ICU* Intensive Care Unit, *GCS* Glasgow Coma Scale

**Fig. 3:**
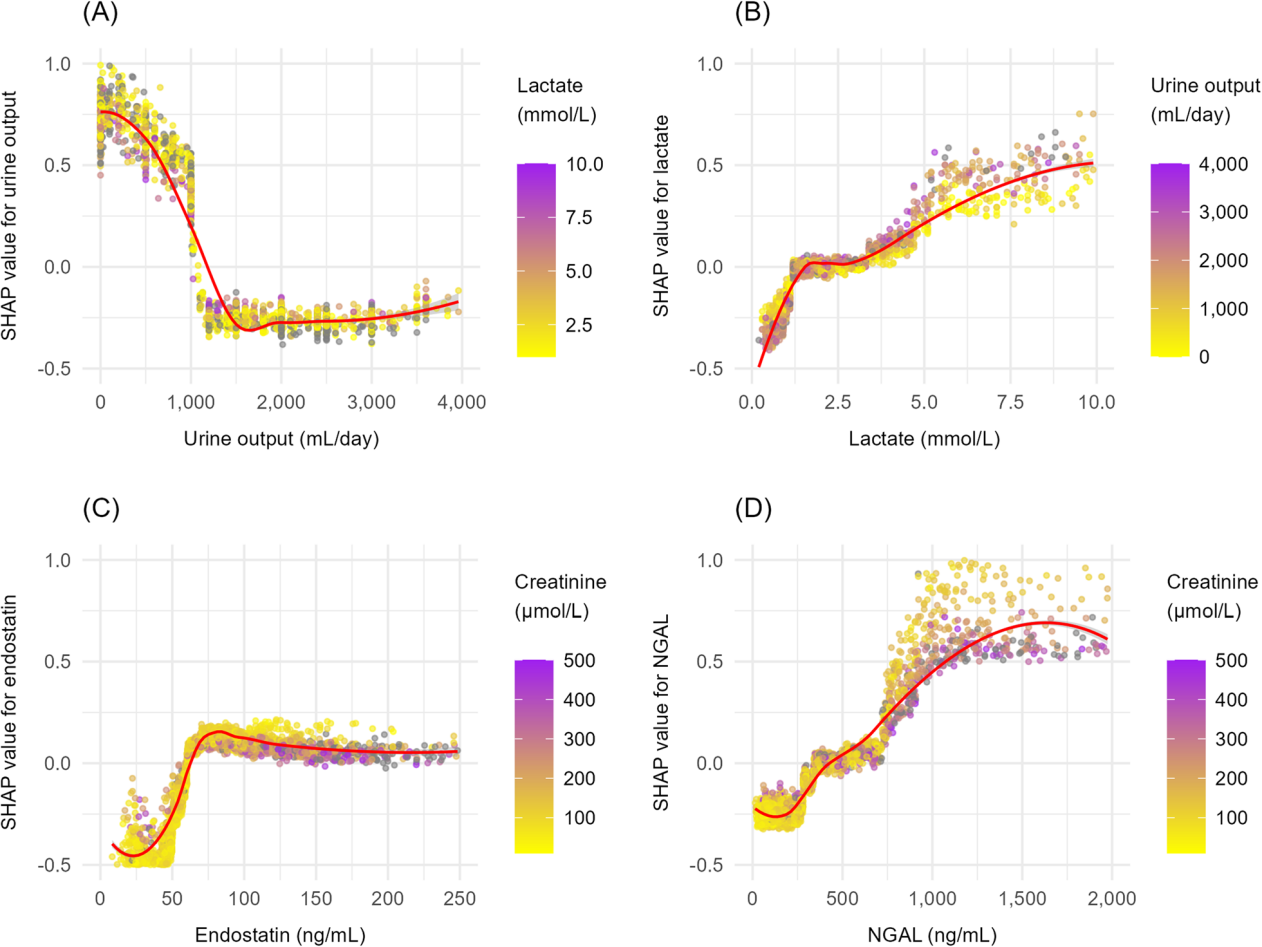
SHapley Additive exPlanations (SHAP) dependence plots for selected key predictors in eXtreme Gradient Boosting (XGBoost) models for AKI (**A**,** B**) and RRT (**C**,** D**). Each point represents a patient, with SHAP value on the *y*-axis indicating each variable’s contribution to the prediction. Variable values are colour-coded from low (yellow) to high (purple). Smoothing curves (red) illustrate the average relationship between variable values and their contributions to the predicted risk. *AKI* Acute Kidney Injury, *RRT* Renal Replacement Therapy, *NGAL* Neutrophil Gelatinase-Associated Lipocalin

See Fig. 2 for SHAP summary plots of the top 10 predictors of new-onset AKI and RRT. See Fig. 3 for SHAP dependence plots of selected key predictors of new-onset AKI and RRT chosen for their non-linear associations or clinical relevance. See Supplementary Figures 1 and 2 for SHAP dependence plots of all the top five SHAP-ranked predictors. See Supplementary Table 1 for the included variables in XAI and logistic regression analyses. See Supplementary Table 2 for the top 10 predictors of new-onset AKI and RRT based on odds ratios from logistic regression analyses, provided as a comparison to the SHAP analyses.

#### New-onset AKI

**Fig. 4:**
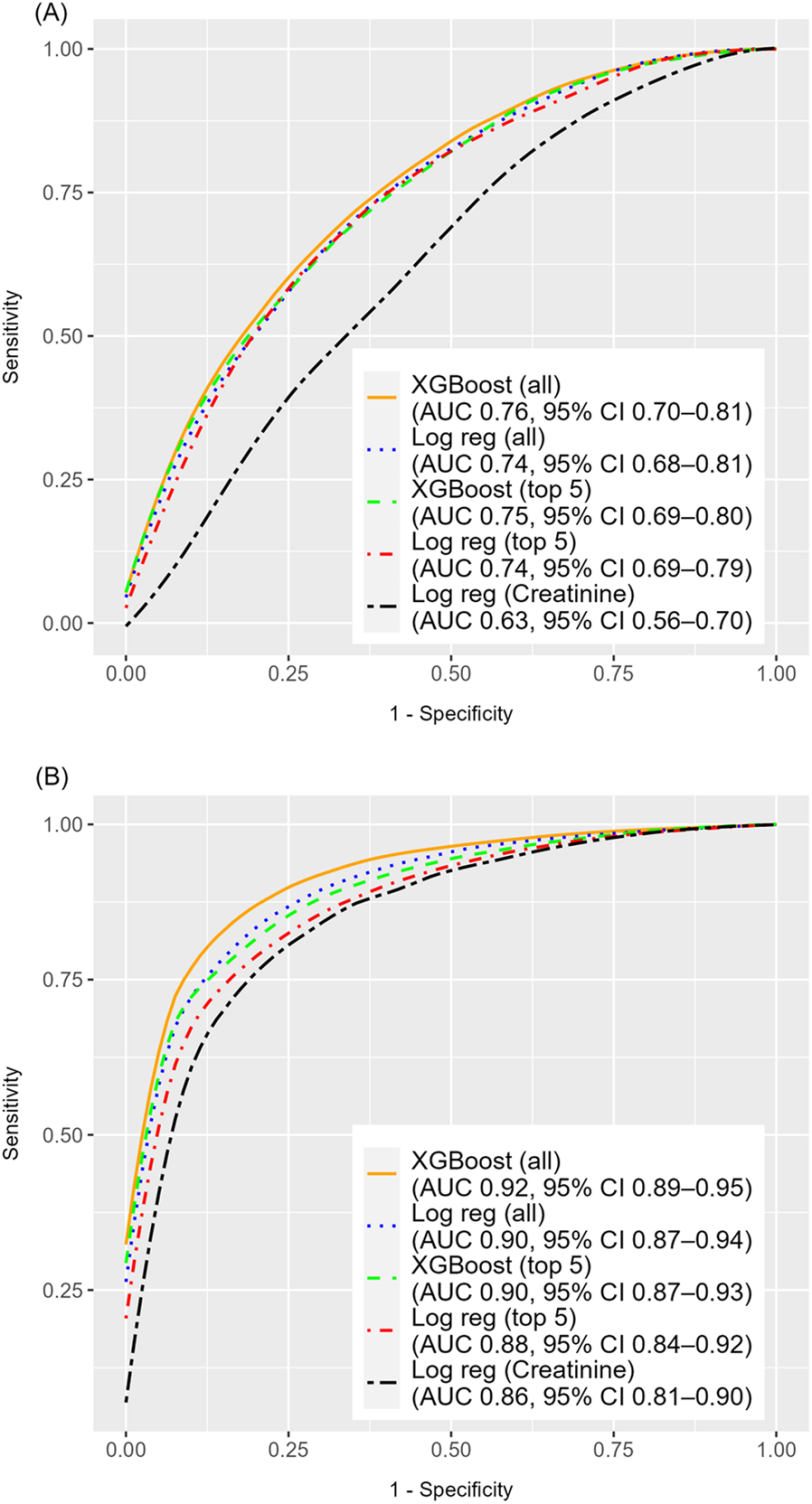
Receiver operating characteristic (ROC) curves for the prediction of new-onset AKI (**A**) and RRT (**B**). Five models were compared: XGBoost and logistic regression with all variables, XGBoost and logistic regression with the top five SHAP-ranked variables, and logistic regression with only creatinine. AUC values represent means, and CIs were derived from repeated cross-validation. Curves represent smoothed averages across validation folds. *XGBoost* eXtreme Gradient Boosting, *Log reg* Logistic regression, *AUC* Area Under the Curve, *CI*, Confidence Interval, *AKI* Acute Kidney Injury, *RRT* Renal Replacement Therapy, *SHAP* SHapley Additive exPlanations

An XGBoost model including all eligible predictor variables demonstrated superior discrimination (mean AUC 0.76, 95% CI 0.70–0.81) compared to a logistic regression model with the same variables (mean AUC 0.74, 95% CI 0.68–0.81; *p* < 0.001). An XGBoost model including only the top five SHAP-ranked variables (urine output, endostatin, baseline creatinine, lactate, and albumin) also outperformed logistic regression using the same variables (mean AUC 0.75, 95% CI 0.69–0.80 vs. 0.74, 95% CI 0.69–0.79; *p* < 0.001). See Fig. 4.

#### RRT

An XGBoost model including all eligible predictor variables demonstrated superior discrimination (mean AUC 0.92, 95% CI 0.89–0.95) compared to a logistic regression model with the same variables (mean AUC 0.90, 95% CI 0.87–0.94; *p* < 0.001). Similarly, an XGBoost model using only the top five SHAP-ranked variables (creatinine, urine output, endostatin, NGAL, and SAPS 3) outperformed logistic regression using the same variables (mean AUC 0.90, 95% CI 0.87–0.93 vs. 0.88, 95% CI 0.84–0.92; *p* < 0.001). See Fig. 4.

## Discussion

This multicentre study identified the most important predictors of new-onset AKI and RRT at ICU admission, including both emerging and established biomarkers, using XGBoost. The most important predictors of new-onset AKI were urine output, endostatin, baseline creatinine, lactate, and albumin. The most important predictors of RRT need were creatinine, urine output, endostatin, NGAL, and SAPS 3. SHAP analysis revealed clinically relevant non-linear relationships, notably for urine output and endostatin. Furthermore, XGBoost outperformed logistic regression in predicting new-onset AKI and RRT, using all available variables or only the top five predictors.

This is the third study to apply XGBoost for AKI prediction in a general ICU cohort, and the first to extend its use to RRT prediction. Previous studies also found that XGBoost was superior to several ML algorithms and logistic regression in predicting AKI. Gao et al. found that the top predictors of AKI were ICU length of stay, creatinine, albumin, electrolytes, blood urea nitrogen, and glucose [6]. The model developed by Zhang et al. identified the top predictors as diuretic use, mechanical ventilation, vasopressor use, age, and antibiotic use [7]. Several factors may explain the differing top predictors compared to the current study. Both previous studies defined AKI solely based on creatinine and lacked access to urine output, which likely limited AKI detection. They included variables obtained at varying timepoints after ICU admission, which limits their applicability at the time of ICU admission. Most importantly, differences in variable inclusion likely also accounted for the divergent findings. While the prior studies were larger, the present study focused specifically on ICU admission and uniquely incorporated emerging biomarkers, offering broader applicability and novel insights into AKI prediction.

Creatinine was the strongest predictor of RRT, but was not among the top 10 predictors of new-onset AKI, underscoring its limitations as an early marker of AKI [2, 3]. In addition, patients with AKI at ICU admission, who likely had elevated creatinine, were excluded from the analysis of new-onset AKI. The strong performance of creatinine in RRT prediction is not surprising, as RRT typically reflects more advanced AKI, allowing sufficient time for creatinine accumulation to occur. Furthermore, creatinine may have introduced bias in RRT prediction, as rising or elevated creatinine likely influenced decisions to initiate RRT.

Urine output emerged as a strong predictor of new-onset AKI, as expected [23, 24]. In contrast to clinical intuition and our findings, urine output has not been identified as a key predictor of RRT in earlier studies [25, 26]. The SHAP dependence plot revealed a steep, non-linear decline in AKI risk with increasing urine output, with an inflexion point around 1500 mL/day, beyond which additional output had minimal impact. While several studies have proposed lowering the KDIGO threshold of 0.5 mL/kg/h, our findings suggest that a higher threshold may better reflect early AKI risk when extrapolated to mL/kg/h [26, 27].

Endostatin was an important predictor of both new-onset AKI and RRT. Although earlier findings were mixed, recent studies have supported its potential as an early biomarker for AKI and possibly also RRT [12, 28–31]. Endostatin is a fragment of collagen XVIII, a basement membrane component found in many tissues, including blood vessels and renal structures. It functions as an angiogenesis inhibitor and is released during extracellular matrix remodelling, with elevated plasma levels likely reflecting microvascular dysfunction, tissue remodelling, and impaired angiogenesis [32, 33]. The SHAP curve for endostatin (Fig. 3A) showed a sharp increase in risk up to around 75 ng/mL, followed by a plateau, suggesting a threshold effect with no added risk at higher levels. Interestingly, the combination of high endostatin and low creatinine exhibited a higher risk of RRT than high endostatin and high creatinine. This could indicate that endostatin captures the early, evolving phase of AKI, when kidney injury has occurred but GFR has not yet declined. In contrast, some patients with elevated creatinine levels may be in a stable, steady-state condition without acute deterioration, making RRT less likely despite high creatinine values.

NGAL has been a promising biomarker for AKI for over two decades, with multiple reviews and meta-analyses supporting its role in predicting both AKI and RRT [11, 34–36]. In our analyses, NGAL ranked as the eighth most important predictor of new-onset AKI and the fourth most important predictor of RRT, findings that are broadly consistent with previous studies. However, several other biomarkers were more influential for both outcomes in this study.

The SHAP curve for lactate (Fig. 3B) showed a rapid rise in AKI risk at low lactate levels (up to approximately 1.4 mmol/L), followed by a brief plateau, and then a renewed, less steep increase beyond approximately 2.5 mmol/L. While most evidence links elevated lactate above the normal range to poor outcomes, our findings suggest that even increases within the low-normal range may carry prognostic significance for AKI [37, 38]. A similar pattern has been reported previously, where rising lactate levels in the low range were associated with higher mortality [39]. Furthermore, dynamic changes in hyperlactataemia have been linked to AKI, and the lactate/albumin ratio has been shown to predict RRT in septic shock [40, 41]. Albumin was the fifth most important predictor of AKI, consistent with a large meta-analysis that showed albumin independently predicts AKI [14]. Bilirubin has also been identified as a predictor of AKI in a previous ML-based study [42].

Cystatin C, a well-studied renal biomarker, was not among the top predictors of AKI and ranked only tenth for RRT, which contrasts with earlier studies [10, 43]. As cystatin C reflects GFR rather than kidney injury, its ability to detect early AKI may be limited. Its predictive value for new-onset AKI may also have been affected by the exclusion of AKI at the time of ICU admission.

This study uniquely integrated emerging biomarkers, routine clinical data, and XAI at the time of ICU admission, enabling a comprehensive evaluation of biomarker utility. The powerful XAI algorithm with XGBoost enabled accurate prediction and interpretation through SHAP values, revealing independent and non-linear associations. The focus on new-onset AKI, with the inclusion of baseline creatinine before ICU admission and exclusion of patients with AKI at ICU admission, is a methodological strength that avoids misclassification and better reflects the potential for early intervention. The resulting models are applicable immediately upon ICU admission, a crucial window for risk stratification and management. Incorporating the dynamics of biomarkers through analyses at several timepoints during the early ICU stay could have further improved predictive performance. However, such an approach would reduce the clinical usefulness of the findings at ICU admission, when early risk stratification and intervention are most critical.

Although the improvements in predictive performance with XGBoost over logistic regression were highly statistically significant for all models, the absolute differences in mean AUC were small and the CIs overlapped. This could suggest that the incremental gain of XGBoost may be of limited clinical relevance at present. Nonetheless, the inclusion of additional promising AKI biomarkers or further optimisation of model hyperparameters could widen the performance gap.

Furthermore, the retrospective design of this study introduces potential bias. Missing data for most variables were limited; however, missing data prevented the inclusion of body weight and BMI in the models and complicated normalisation of urine output. Missing baseline creatinine values may have led to overestimation of AKI. To improve data reliability and meet the KDIGO time criterion for oliguria ($\ge$ 6 h), we chose not to apply the KDIGO urine output criteria for AKI at ICU admission [17]. Consistent with previous studies, we found that urine output data obtained on ICU admission are frequently unreliable or missing [44, 45]. While this may have led to under-recognition of AKI present on ICU admission, our definition of new-onset AKI was otherwise strict and included both creatinine and urine output on ICU days 1 and 2. Although repeated cross-validation was used to mitigate overfitting, the absence of an independent test set may also limit the generalisability of our findings. In addition, the study was conducted in ICUs within a single geographic region, which may further restrict external validity. Future external validation and prospective studies are, therefore, warranted.

This study supports the development of XGBoost-based risk scores to guide early AKI risk stratification and future clinical trials, potentially improving outcomes in the ICU. The strong performance of biomarkers such as endostatin and NGAL highlights their promise in identifying early AKI, helping to address the persistent challenge of late AKI detection.

## Conclusions

This multicentre study identifies the most important predictors of early new-onset AKI and the need for RRT at ICU admission using XAI, highlighting both emerging biomarkers, such as endostatin and NGAL, and established biomarkers, including lactate and albumin. XGBoost enhances the prediction of AKI and RRT by integrating routine clinical data with emerging biomarkers, thereby revealing clinically important non-linear relationships. External validation and prospective studies assessing the clinical integration of such models are warranted.

## Supplementary Information

Below is the link to the electronic supplementary material.

Supplementary file1 (PDF 10858 KB)

## Data Availability

The data sets generated and analysed during the current study are not publicly available due to limitations in the ethical approval of the study and data management policies of Region Skåne. However, they are available from the corresponding author on request.

## References

[1] Hoste EAJ, Kellum JA, Selby NM, Zarbock A, Palevsky PM, Bagshaw SM et al (2018) Global epidemiology and outcomes of acute kidney injury. Nat Rev Nephrol 14(10):607–625. 10.1038/s41581-018-0052-0 30135570 10.1038/s41581-018-0052-0

[2] Waikar SS, Bonventre JV (2009) Creatinine kinetics and the definition of acute kidney injury. J Am Soc Nephrol 20(3):672–679. 10.1681/ASN.2008070669 19244578 10.1681/ASN.2008070669 PMC2653692

[3] Ostermann M, Legrand M, Meersch M, Srisawat N, Zarbock A, Kellum JA (2024) Biomarkers in acute kidney injury. Ann Intensive Care 14(1):145. 10.1186/s13613-024-01360-9 39279017 10.1186/s13613-024-01360-9 PMC11402890

[4] Chen T, Guestrin C. XGBoost: a scalable tree boosting system. In: Proceedings of the 22nd ACM SIGKDD international conference on knowledge discovery and data mining. ACM; 2016. p. 785–794

[5] Lundberg SM, Lee SI (2017) A unified approach to interpreting model predictions. Adv Neural Inf Process Syst 30:4765–4774

[6] Gao W, Wang J, Zhou L, Luo Q, Lao Y, Lyu H et al (2022) Prediction of acute kidney injury in ICU with gradient boosting decision tree algorithms. Comput Biol Med 140:105097. 10.1016/j.compbiomed.2021.105097 34864304 10.1016/j.compbiomed.2021.105097

[7] Zhang L, Li M, Wang C, Zhang C, Wu H (2025) Prediction of acute kidney injury in intensive care unit patients based on interpretable machine learning. Digit Health 11:20552076241311172. 10.1177/20552076241311173 39777058 10.1177/20552076241311173 PMC11705319

[8] Frigyesi A, Lengquist M, Johnsson P, Lybeck A, Spångfors M, Levin H et al (2023) The Swecrit Biobank, associated clinical registries, and machine learning (artificial intelligence) improve critical care knowledge. Läkartidningen 120:23078 37846149

[9] Von Elm E, Altman DG, Egger M, Pocock SJ, Gøtzsche PC, Vandenbroucke JP (2007) The Strengthening the Reporting of Observational Studies in Epidemiology (STROBE) statement: guidelines for reporting observational studies. Ann Intern Med 147(8):573–577. 10.7326/0003-4819-147-8-200710160-00010 17938396 10.7326/0003-4819-147-8-200710160-00010

[10] Yong Z, Pei X, Zhu B, Yuan H, Zhao W (2017) Predictive value of serum cystatin C for acute kidney injury in adults: a meta-analysis of prospective cohort trials. Sci Rep 7:41012. 10.1038/srep41012 28112204 10.1038/srep41012 PMC5253621

[11] Xu C, Lin S, Mao L, Li Z (2022) Neutrophil gelatinase-associated lipocalin as predictor of acute kidney injury requiring renal replacement therapy: a systematic review and meta-analysis. Front Med 9:859318. 10.3389/fmed.2022.859318 10.3389/fmed.2022.859318 PMC9533127 36213627

[12] Koozi H, Engström J, Larsson A, Spångfors M, Friberg H, Frigyesi A (2025) Plasma endostatin and its association with new-onset acute kidney injury in critical care. J Intensive Care 13(1):48. 10.1186/s40560-025-00820-z 40898305 10.1186/s40560-025-00820-z PMC12403487

[13] Sathe NA, Mostaghim A, Barnes E, O’Connor NG, Sahi SK, Sakr SS et al (2023) Biomarker signatures of severe acute kidney injury in a critically ill cohort of COVID-19 and Non-COVID-19 acute respiratory illness. Crit Care Explor 5(7):e0945. 10.1097/CCE.0000000000000945 37457915 10.1097/CCE.0000000000000945 PMC10348733

[14] Wiedermann CJ, Wiedermann W, Joannidis M (2010) Hypoalbuminemia and acute kidney injury: a meta-analysis of observational clinical studies. Intensive Care Med 36(10):1657–1665. 10.1007/s00134-010-1928-z. Erratum in: Intensive Care Med. 2021;47(2):262. https://doi.org/10.1007/s00134-020-06268-z. 20517593 10.1007/s00134-010-1928-z PMC7728653

[15] Lengquist M, Sundén-Cullberg J, Hyllner S, Koozi H, Larsson A, Mellhammar L et al (2025) Calprotectin as a sepsis diagnostic marker in critical care: a retrospective observational study. Sci Rep 15(1):15529. 10.1038/s41598-025-95420-0 40319081 10.1038/s41598-025-95420-0 PMC12049440

[16] Inker LA, Eneanya ND, Coresh J, Tighiouart H, Wang D, Sang Y et al (2021) New creatinine- and cystatin C-based equations to estimate GFR without race. N Engl J Med 385(19):1737–1749. 10.1056/NEJMoa2102953 34554658 10.1056/NEJMoa2102953 PMC8822996

[17] Khwaja A (2012) KDIGO clinical practice guidelines for acute kidney injury. Nephron Clin Pract 120(4):c179-184. 10.1159/000339789 22890468 10.1159/000339789

[18] Moreno RP, Metnitz PG, Almeida E, Jordan B, Bauer P, Campos RA et al (2005) SAPS 3—from evaluation of the patient to evaluation of the intensive care unit. Part 2: development of a prognostic model for hospital mortality at ICU admission. Intensive Care Med 31(10):1345–1355. 10.1007/s00134-005-2763-5 16132892 10.1007/s00134-005-2763-5 PMC1315315

[19] Singer M, Deutschman CS, Seymour CW, Shankar-Hari M, Annane D, Bauer M et al (2016) The third international consensus definitions for sepsis and septic shock (Sepsis-3). JAMA 315(8):801–810. 10.1001/jama.2016.0287 26903338 10.1001/jama.2016.0287 PMC4968574

[20] Mellhammar L, Elén S, Ehrhard S, Bouma H, Ninck L, Muntjewerff E et al (2022) New, useful criteria for assessing the evidence of infection in sepsis research. Crit Care Exp 4(5):e0697. 10.1097/CCE.0000000000000697 10.1097/CCE.0000000000000697 PMC9116943 35620771

[21] R Core Team. R: A language and environment for statistical computing. Vienna, Austria: R Foundation for Statistical Computing. https://www.R-project.org/. Accessed 15 Feb 2025

[22] DeLong ER, DeLong DM, Clarke-Pearson DL (1988) Comparing the areas under two or more correlated receiver operating characteristic curves: a nonparametric approach. Biometrics 44(3):837–845. 10.2307/2531595 3203132

[23] Malbrain MLNG, Tantakoun K, Zara AT, Ferko NC, Kelly T, Dabrowski W (2024) Urine output is an early and strong predictor of acute kidney injury and associated mortality: a systematic literature review of 50 clinical studies. Ann Intensive Care 14(1):110. 10.1186/s13613-024-01342-x 38980557 10.1186/s13613-024-01342-x PMC11233478

[24] Yamamoto R, Yamakawa K, Yoshizawa J, Kaito D, Umemura Y, Homma K et al (2025) Urine output and development of acute kidney injury in sepsis: a multicenter observational study. J Intensive Care Med 40(2):191–199. 10.1177/08850666241268390 39094594 10.1177/08850666241268390

[25] Koziolek MJ, Datta RR, Mattes H, Jung K, Heise D, Streich JH et al (2012) Predictors of renal replacement therapy in acute kidney injury. Nephron Extra 2(1):247–255. 10.1159/000342257 23599703 10.1159/000342257 PMC3567877

[26] Klein SJ, Lehner GF, Forni LG, Joannidis M (2018) Oliguria in critically ill patients: a narrative review. J Nephrol 31(6):855–862. 10.1007/s40620-018-0539-6 30298272 10.1007/s40620-018-0539-6 PMC6244549

[27] Md Ralib A, Pickering JW, Shaw GM, Endre ZH (2013) The urine output definition of acute kidney injury is too liberal. Crit Care 17(3):R112. 10.1186/cc12784 23787055 10.1186/cc12784 PMC4056349

[28] Mårtensson J, Jonsson N, Glassford NJ, Bell M, Martling CR, Bellomo R et al (2016) Plasma endostatin may improve acute kidney injury risk prediction in critically ill patients. Ann Intensive Care 6(1):6. 10.1186/s13613-016-0108-x 26762504 10.1186/s13613-016-0108-x PMC4712179

[29] Mårtensson J, Vaara ST, Pettilä V, Ala-Kokko T, Karlsson S, Inkinen O et al (2017) Assessment of plasma endostatin to predict acute kidney injury in critically ill patients. Acta Anaesthesiol Scand 61(10):1286–1295. 10.1111/aas.12988 28857121 10.1111/aas.12988

[30] Ruge T, Larsson A, Lipcsey M, Tydén J, Johansson J, Eriksson M (2021) A comparison between endostatin and conventional biomarkers on 30 day mortality and renal replacement therapy in unselected intensive care patients. Biomedicines. 10.3390/biomedicines9111603 34829832 10.3390/biomedicines9111603 PMC8615500

[31] Koozi H, Engström J, Zwawi A, Spångfors M, Didriksson I, Larsson A et al (2025) Plasma endostatin at intensive care admission is independently associated with acute kidney injury, dialysis, and mortality in COVID-19. Intensive Care Med Exp 13(1):42. 10.1186/s40635-025-00748-6 40178654 10.1186/s40635-025-00748-6 PMC11968582

[32] Marneros AG, Olsen BR (2005) Physiological role of collagen XVIII and endostatin. FASEB J 19(7):716–728 15857886 10.1096/fj.04-2134rev

[33] Li M, Popovic Z, Chu C, Krämer BK, Hocher B (2021) Endostatin in renal and cardiovascular diseases. Kidney Dis (Basel) 7(6):468–481. 10.1159/000518221 34901193 10.1159/000518221 PMC8613550

[34] Mishra J, Ma Q, Prada A, Mitsnefes M, Zahedi K, Yang J et al (2003) Identification of neutrophil gelatinase-associated lipocalin as a novel early urinary biomarker for ischemic renal injury. J Am Soc Nephrol 14(10):2534–2543. 10.1097/01.ASN.0000088027.54400.C6 14514731 10.1097/01.asn.0000088027.54400.c6

[35] Haase M, Bellomo R, Devarajan P, Schlattmann P, Haase-Fielitz A, NGALMeta-analysis Investigator Group (2009) Accuracy of neutrophil gelatinase-associated lipocalin (NGAL) in diagnosis and prognosis in acute kidney injury: a systematic review and meta-analysis. Am J Kidney Dis 54(6):1012–1024. 10.1053/j.ajkd.2009.07.020 19850388 10.1053/j.ajkd.2009.07.020

[36] Devarajan P (2010) Review: Neutrophil gelatinase-associated lipocalin: a troponin-like biomarker for human acute kidney injury. Nephrology (Carlton) 15(4):419–428. 10.1111/j.1440-1797.2010.01317.x 20609093 10.1111/j.1440-1797.2010.01317.x

[37] Vincent JL, Quintairos e Silva A, Couto LJ, Taccone FS. (2016) The value of blood lactate kinetics in critically ill patients: a systematic review. Crit Care 20(1):257. 10.1186/s13054-016-1403-5 27520452 10.1186/s13054-016-1403-5 PMC4983759

[38] Rabello Filho R, Rocha LL, Corrêa TD, Pessoa CM, Colombo G, Assunção MS (2016) Blood lactate levels cutoff and mortality prediction in sepsis-time for a reappraisal? A retrospective cohort study. Shock 46(5):480–485. 10.1097/SHK.0000000000000667 27380535 10.1097/SHK.0000000000000667 PMC5058781

[39] Andersson P, Frigyesi A (2018) Lactate improves SAPS 3 prognostication. Acta Anaesthesiol Scand 62(2):220–225. 10.1111/aas.13033 29124742 10.1111/aas.13033

[40] Fang Y, Shen X, Dou A, Xie H, Zhang Y, Zhang Y et al (2025) Utilization of lactate trajectory models for predicting acute kidney injury and mortality in patients with hyperlactatemia: insights across three independent cohorts. Ren Fail 47(1):2474205. 10.1080/0886022X.2025.2474205 40074720 10.1080/0886022X.2025.2474205 PMC11905305

[41] Shadvar K, Nader-Djalal N, Vahed N, Sanaie S, Iranpour A, Mahmoodpoor A et al (2022) Comparison of lactate/albumin ratio to lactate and lactate clearance for predicting outcomes in patients with septic shock admitted to intensive care unit: an observational study. Sci Rep 12(1):13047. 10.1038/s41598-022-14764-z 35906231 10.1038/s41598-022-14764-z PMC9338032

[42] Chen Y, Feng F, Li M, Chang X, Wei B, Dong C (2019) Development of a risk stratification-based model for prediction of acute kidney injury in critically ill patients. Medicine (Baltimore) 98(33):e16867. 10.1097/MD.0000000000016867 31415421 10.1097/MD.0000000000016867 PMC6831442

[43] Bagshaw SM, Bellomo R (2010) Cystatin C in acute kidney injury. Curr Opin Crit Care 16(6):533–539. 10.1097/MCC.0b013e32833e8412 20736828 10.1097/MCC.0b013e32833e8412

[44] Wilson FP, Martin M, Yamamoto Y, Partridge C, Moreira E, Arora T et al (2021) Electronic health record alerts for acute kidney injury: multicenter, randomized clinical trial. BMJ 372:m4786. 10.1136/bmj.m4786 33461986 10.1136/bmj.m4786 PMC8034420

[45] Hasidim AA, Klein MA, Ben Shitrit I, Einav S, Ilan K, Fuchs L (2025) Toward the standardization of big datasets of urine output for AKI analysis: a multicenter validation study. Sci Rep 15(1):20009. 10.1038/s41598-025-95535-4 40481220 10.1038/s41598-025-95535-4 PMC12144275

