# Supplementary material for: Explainable AI identifies key biomarkers for acute kidney injury prediction in the ICU
Source: Intensive Care Med Exp. 2025 Oct 22;13:106. doi: 10.1186/s40635-025-00816-x (PMC12545979; doi:10.1186/s40635-025-00816-x)
Supplement: Supplementary file 1 — Supplementary file1 (PDF 10858 KB) [file 40635_2025_816_MOESM1_ESM.pdf]

Explainable AI identifies key biomarkers for acute kidney injury prediction in the ICU

Hazem Koozi[1,2*], Jonas Engström[1,2], Hans Friberg[1,3], Attila Frigyesi[1,4]

[1]Department of Clinical Sciences, Anaesthesiology and Intensive Care, Lund University, SE-22185, Lund, Sweden.
[2]Department of Anaesthesia and Intensive Care, Skåne University Hospital, SE-29133, Kristianstad, Sweden.
[3]Department of Intensive and Perioperative Care, Skåne University Hospital, SE-20502, Malmö, Sweden.
[4]Department of Intensive and Perioperative Care, Skåne University Hospital, SE-22185, Lund, Sweden.

*Corresponding author(s). E-mail(s): hazem.koozi@med.lu.se;
Contributing authors: jonas.engstrom@med.lu.se;
hans.friberg@med.lu.se; attila.frigyesi@med.lu.se;

Supplementary material

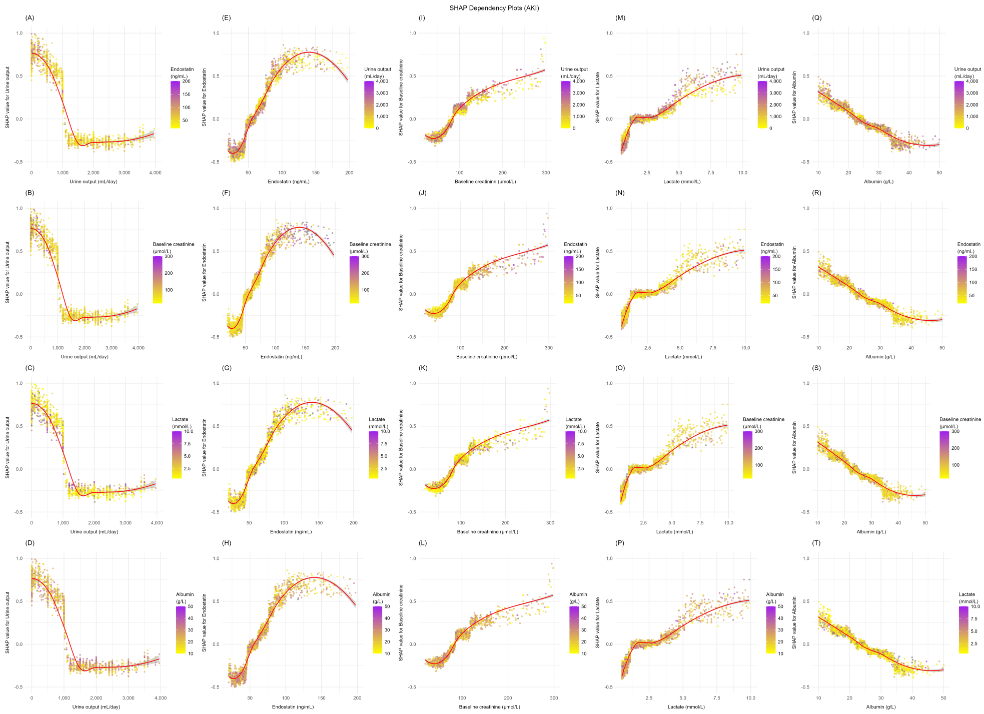

Supplementary Fig. 1 SHapley Additive exPlanations (SHAP) dependence plots for key predictors in an eXtreme Gradient Boosting (XGBoost) model for acute kidney injury. Each point represents a patient, with SHAP value on the y-axis indicating the variable's impact on the prediction. Variable values are color-coded from low (yellow) to high (purple). Smoothing curves (red) illustrate associations between variable value and SHAP contribution.

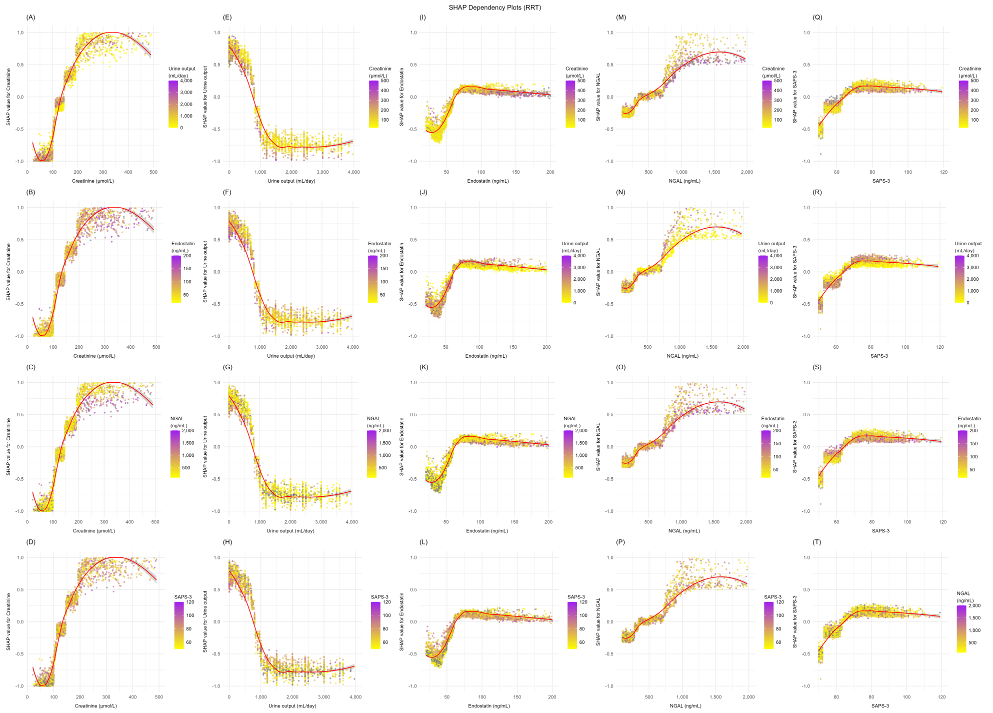

Supplementary Fig. 2 SHapley Additive exPlanations (SHAP) dependence plots for key predictors in an eXtreme Gradient Boosting (XGBoost) model for renal replacement therapy. Each point represents a patient, with SHAP value on the y-axis indicating the variable's impact on the prediction. Variable values are color-coded from low (yellow) to high (purple). Smoothing curves (red) illustrate associations between variable value and SHAP contribution. *NGAL* Neutrophil Gelatinase-Associated Lipocalin. *SAPS-3* Simplified Acute Physiology Score 3.

Supplementary Table 1 Predictor variables included in analyses of new-onset AKI and RRT.

Variable	Type
**General characteristics**	
Age	Continuous
Sex	Binary
Sepsis-3	Binary
Septic shock	Binary
Sepsis LMCI	Binary
Nosocomial infection	Binary
Respiratory infection	Binary
Antibiotic use	Binary
Cardiac arrest	Binary
Trauma	Binary
Elective surgery	Binary
Emergency surgery	Binary
Time before ICU	Continuous
Mechanical ventilation/NIV	Binary
**Comorbidities**	
Immunosuppression	Binary
Cancer	Binary
Haematological cancer	Binary
Cirrhosis	Binary
Chronic heart failure	Binary
AIDS	Binary
**Illness severity**	
SAPS 3	Continuous
PaO_2	Continuous
PaO_2/FiO_2	Continuous
Heart rate	Continuous
Systolic blood pressure	Continuous
Mean arterial pressure	Continuous
Cardiovascular SOFA score	Ordinal
Vasopressor use	Binary
Noradrenaline	Ordinal
Adrenaline	Ordinal
Vasopressin	Binary
Dobutamine	Binary
Levosimendan	Binary
Urine output	Continuous
Glasgow Coma Scale	Ordinal
Body temperature	Continuous
AKI*	Binary
**Laboratory values**	
Baseline creatinine	Continuous
Creatinine	Continuous
Cystatin C	Continuous
Platelet count	Continuous
White blood cell count	Continuous
Lactate	Continuous
Bilirubin	Continuous
C-reactive protein	Continuous
Albumin	Continuous
NGAL	Continuous
ICAM-1	Continuous
VCAM-1	Continuous
Calprotectin	Continuous
Endostatin	Continuous
pH	Continuous

Variables are at ICU admission. *Only included in RRT analysis. *AKI* Acute Kidney Injury, *RRT* Renal Replacement Therapy, *LMCI* Linder-Mellhammar Criteria of Infection, *ICU* Intensive Care Unit, *NIV* Non-invasive Ventilation, *AIDS* Acquired Immunodeficiency Syndrome, *SAPS* Simplified Acute Physiology Score, PaO_2, Arterial Partial Pressure of Oxygen, FiO_2 Fraction of Inspired Oxygen (%), *SOFA* Sequential Organ Failure Assessment, *NGAL* Neutrophil Gelatinase-Associated Lipocalin, *ICAM-1* Intercellular Adhesion Molecule 1, *VCAM-1* Vascular Cell Adhesion Molecule 1.

Supplementary Table 2 Logistic regression results for the top 10 predictors of new-onset AKI and RRT ranked by OR.

Variable	OR	95% CI
**New-onset AKI**		
Baseline creatinine	1.4	1.2–1.7
Endostatin	1.4	1.3–1.6
Cardiovascular SOFA	1.4	0.95–2.0
Lactate	1.3	1.2–1.5
SAPS 3	1.3	1.0–1.6
Bilirubin	1.2	1.1–1.4
NGAL	1.2	1.0–1.3
Antibiotic use	1.2	1.0–1.3
GCS	1.1	0.98–1.3
Adrenaline	1.1	1.0–1.3
**RRT**		
Creatinine	2.6	2.1–3.2
NGAL	1.4	1.2–1.7
GCS	1.4	1.2–1.7
Time before ICU	1.4	1.2–1.6
SAPS 3	1.4	1.1–1.8
Lactate	1.4	1.2–1.6
MAP	1.3	1.1–1.5
Sepsis LMCI	1.3	1.1–1.5
Noradrenaline	1.2	0.90–1.7
Bilirubin	1.2	1.0–1.4

Continuous variables were z-scored before analyses. ORs are per 1 standard deviation increase for continuous variables. *AKI* Acute Kidney Injury, *RRT* Renal Replacement Therapy, *OR* Odds Ratio, *CI* Confidence Interval, *SOFA* Sequential Organ Failure Assessment, *SAPS* Simplified Acute Physiology Score, *NGAL* Neutrophil Gelatinase-Associated Lipocalin, *GCS* Glasgow Coma Scale, *ICU* Intensive Care Unit, *MAP* Mean Arterial Pressure, *LMCI* Linder-Mellhammar Criteria of Infection.
